# The contemporary economic breakdown of Pakistan and its consequences on the mental health of inflation‐stricken nation

**DOI:** 10.1002/hsr2.70094

**Published:** 2024-09-23

**Authors:** Tasmiyah Siddiqui, Ayesha Saadat, Rooja Zubair, Shafaq Taseen

**Affiliations:** ^1^ Department of Internal Medicine Dow University of Health Sciences Karachi Pakistan; ^2^ Department of Medicine Karachi Medical and Dental College Karachi Pakistan

## TRANSPARENCY STATEMENT

We, the authors of this study, affirm that this manuscript is an honest, accurate, and transparent account of the study being reported, that no important aspects of the study have been omitted, and that any discrepancies from the study as planned (and, if relevant, registered) have been explained.

Dear Editor,

Over the past seven decades, Pakistan has grappled with recurring episodes of political instability that have invariably led to severe economic and financial crises in the country. Recent months, however, have brought unprecedented challenges to our nation's economic stability. A dramatic surge in fuel prices, soaring electricity bills, abrupt hikes in sales taxes, and the relentless impact of the COVID‐19 pandemic have coalesced to create a perfect storm. With a population of approximately 231 million, Pakistan is teetering on the brink of what could be a historically catastrophic economic breakdown. The nation's mental health is now at stake, given the sharp spikes in inflation and the resulting financial hardships faced by its citizens.

Many studies have shown positive association between financial constraints and onset of mental health problems.^1^ Similarly, multiple suicide cases of young people have been reported in the past few months due to poverty, joblessness, and inflation in different cities of Pakistan.^2^ These dire circumstances are particularly concerning given that mental healthcare services remain limited, and mental health concerns are often stigmatized, preventing individuals from seeking help.

The economic crisis extends its damaging effects to vulnerable populations, including children growing up in impoverished households. Such circumstances can give rise to a host of emotional, social, and intellectual problems that may hinder their transition into productive adulthood. Adding to the complexity, nearly 40% of Pakistan's population lacks health insurance, which creates another significant barrier to accessing mental health services.^3^

Although, in Pakistan, mental health services are included in the Sehat Sahulat Program, which is a health insurance scheme for underprivileged citizens. However, it's important to note that mental illness is often not fully covered by insurance under this program, and government spending is considered the major source of financing for affordable healthcare. The budget allocation for mental health services is crucial, and there is a need for a particular focus on low‐resource districts to avoid patients and their families having to travel to tertiary care hospitals in better‐resourced districts of Pakistan.^4^ With the recent skyrocketing inflation and the contemporary wave of political turmoil in the country, the GDP rates are dropping drastically day to day, with a figure of 5% in the last few years and the foreign reserves of the country are also replenishing expeditiously. According to an economic survey report 2023, the value of the rupee has been depreciating persistently at a rate of 24% since the year‐to‐date against the rate of the dollar, which is appraising up to 17%, the fact which is convincing International Monetary Funds to continue the legacy of their grants and loans to the country making the situation further complicated.^5^, ^6^ Pakistan is facing the worst economic crisis in history up until now with the inflation rate being 24.76%, which is affecting the whole nation terribly.^6^ According to different economic surveys, the rate of joblessness has been reported at up to 31%, which creates a sense of uncertainty and hopelessness among the well‐educated and qualified younger bloodline of the nation and is correlated to the augmenting suicidal rates. Each year 130,000–270,000 people commit suicide in Pakistan, and the leading cause of these suicidal deaths is the lack of health facilities provided to the traumatized people of the nation. These cases are more prevalent in the younger generation, which forms a major part, that is, 65% of the country's population.^7^ The percentage of different mental health‐related disorders, including depression and anxiety, is reported at around 10%–16%, and the main culprit behind this entire fiasco is narrated as the frequent change of political regime in the country, the last wave of devastating floods, and the deleterious effects of COVID‐19.^6^

Nevertheless, to truly understand this catastrophe one must examine the cultural features that shape mental health perceptions and behavior regarding seeking treatment. In Pakistan, the culture usually discourages people from dealing with mental health conditions. The traditional values view mental illness as an indication of personal weakness or spiritual problem necessitating religious rituals and family support instead of professional involvement.^8^ Furthermore, women's access to mental health services can be hampered by gender roles and expectations which tend to prioritize their domestic duties over their own welfare. Moreover, Pakistani society is collectivist in nature meaning psychological well‐being is influenced by community and familial interdependence both positively and negatively. To deal with the mental health effects of the financial crisis in Pakistan, specific policies must be put in place and active community participation should be ensured. It is important to act proactively, but much of research conducted recently do not provide exact context‐specific recommendations on how these can be achieved. This will help address these problems and reduce psychological impacts from economic challenges. All the pivotal steps needed to counteract such a devastated situation are underscored below:

1. Develop targeted financial aid and subsidies for mental health care, specifically directed toward disadvantaged and vulnerable communities, to reduce financial barriers. The expansion of microfinance initiatives will make it possible for people to obtain economic stability as well as cope with stress coming from uncertainty related to their finances. Also, the government must provide grants and introduce policies with a view of giving relief as well as rehabilitating the affected citizens.
2. Providing basic training in primary healthcare facilities for mental healthcare would assist in early identification and intervention.
3. Use task shifting procedures that educate nonprofessionals on delivering mental help under the guidance of trained professionals. Requesting assistance from global practitioners could be employed to determine effective approaches in places such as Bangladesh or India about this problem area.
4. Embedding mental health teachings into school syllabuses by using media, internet platforms, and community events to foster lasting strength and consciousness among young people for the purpose of early intervention and mental health promotion.
5. Developing programs meant for increasing employment opportunities especially in mental healthcare support services sector to get rid of unemployment together with the scarcity of mental health professionals
6. Engage community leaders and influencers in advocating for mental health initiatives to foster community backing and participation. Collaborate with NGOs and CBOs to initiate projects which are tailored according to the unique needs and circumstances of the local community aimed at creating confidence and hope among already stressed population thereby discouraging people from taking extreme measures. Establish support systems within communities where people can share stories, coping strategies, challenges, and promote unity while building resilience.
7. Moreover, mental health experts play an important role in reducing the deteriorating mental health of our inflation‐stricken nation. Active engagement with survivors, providing coping mechanisms, engaging in meaningful activities, and directing them on sleep patterns, dietary pattern constitutes some of key aspects to be upheld toward promoting mental health therapy.

Additionally, it is significant that medical coverage and psychiatric services are within an arm's reach to help ameliorate the economic downturn effects on psychological well‐being.

## AUTHOR CONTRIBUTIONS

**Tasmiyah Siddiqui**: Conceptualization; methodology; validation; writing—review and editing. **Ayesha Saadat**: Conceptualization; methodology; visualization; writing—original draft; writing—review and editing. **Rooja Zubair**: Conceptualization; methodology; writing—original draft; writing—review and editing; validation. **Shafaq Taseen**: Writing—review and editing; writing—original draft; validation; conceptualization.

## CONFLICT OF INTEREST STATEMENT

The authors declare no conflict of interest.

## Data Availability

Data sharing is not applicable to this article as no datasets were generated or analyzed during the current study.
